# Impact of the Dam Construction on the Downstream Thermal Conditions of the Yangtze River

**DOI:** 10.3390/ijerph17082973

**Published:** 2020-04-24

**Authors:** Tianfu He, Yun Deng, Youcai Tuo, Yanjing Yang, Naisheng Liang

**Affiliations:** State Key Laboratory of Hydraulics and Mountain River Engineering, Sichuan University, Chengdu 610065, China; htf853835664@163.com (T.H.); 13281109119@163.com (Y.Y.); 2017323060027@stu.scu.edu.cn (N.L.)

**Keywords:** Yangtze River, multiple dams, water temperature, cumulative impact, Chinese sturgeon

## Abstract

Water temperature is an important factor in aquatic environments. Dam construction, especially the construction of multiple dams in rivers, can greatly affect the downstream water temperature. Several dams, including Wudongde, Baihetan, Xiluodu, Xiangjiaba, Three Gorges, and Gezhouba, have been constructed between Panzhihua and Yichang along the Yangtze River. The aim of this paper was to quantify the impact of these dams on the water temperature downstream. One-dimensional and two-dimensional models were used to simulate the water temperatures, and the results showed that the dams had different cumulative effects on it. For example, in January, after the construction of the Xiangjiaba and Xiluodu dams, the discharge water temperature of Xiangjiaba was 3 °C higher than the natural conditions, and after the construction of the Baihetan and Wudongde dams was completed, it increased by a further 2 °C. The natural river ran over 416 km with no dams from the Xiangjiaba dam to the Cuntan Station. With the influence of climate and tributary inflow, the impact of upstream dams on the water temperature was mitigated by more than 48% at Cuntan Station, displaying a recovery. It seemed that the cumulative effects of dams on the discharge water temperature of the Three Gorges decreased with the increase in the upstream storage capacity from March to May, and the construction of dams even had a negative effect. From September to February of the next year, the cumulative effects increased with the increase of the upstream storage capacity, but only the total storage capacity until a certain level, where no further impact was observed.

## 1. Introduction

Currently, many rivers are fragmented by dams, especially large rivers. Dams play important roles in flood control, electricity generation, irrigation, and other processes [1,2,3,4]. In addition to obvious benefits, the construction of dams threatens river ecosystems [5], such as changing hydraulic conditions [6,7], promoting eutrophication, blocking fish migration routes [8,9], and reducing species diversity [10,11]. Additionally, the construction of dams can change the water temperature in a basin. Changes in water temperature may negatively impact the river ecosystems, and as the number of dams increases, this negative impact may accumulate [12,13,14].

Water temperature is an important ecological factor in habitats [15,16,17]. Some fish species are very sensitive to water temperature; they spawn only when the water temperature reaches a certain range [18,19]. Stratification usually occurs in reservoirs, especially large reservoirs [20,21]. The discharge water temperature is usually significantly different from that before the dam construction [22]. Recently, the negative effects of dam construction on the water temperature of downstream rivers have attracted widespread attention. A few examples of these effects can be seen at the Flaming Gorge Dam, the Gathright Dam, and the Shasta Dam in the United States, and the Burrendong Dam and the Keepit Dam in Australia [23,24,25]. Dam construction has been found to lead to changes in the thermal regime [26]. In many rivers, researchers pay attention to the fact that the discharge water temperature of a reservoir is lower than that before the construction of the dam in spring and summer. The variations in the thermal regime modify habitat conditions and biological communities [24]. For example, in some cases, cold-adapted fish species have been found to displace native species [27]. In addition, the discharge water temperature of a reservoir may be higher than that before dam construction. This impact may also affect the ecology in the river. For example, some species spawn only when the water temperature is below a certain level [28,29,30,31,32].

Multiple dams are usually constructed in rivers, and the dams may produce a cumulative effect on downstream water temperature [16]. Liu L.F.’s research results showed that the higher the degree of hydropower development, the greater the cumulative impact on the river water temperature, in the Lancang River Basin. The cumulative impact of five dams is greater than that of three dams, and larger dams contribute more to the cumulative effect than smaller dams [12]. The same trend has been observed in other rivers [14]; as the number of dams increases, the accumulation effect on water temperature becomes more obvious.

The Yangtze River is one of the largest rivers in the world, and it is rich in aquatic species. Four dams have been built on this river, and two dams are under construction, with more dams possibly planned further upstream in the future. In 2012, Liang, R.F. used a two-dimensional water temperature model to predict the accumulative impact of the dams (including Xiangjiaba, Xiluodu, Baihetan, and Wudongde) on the water temperature [16]. According to the research results, the construction of the dams had a cumulative effect on the discharge water temperature of the Xiangjiaba. The thermal conditions further downstream of the Xiangjiaba dam were not analyzed. There are many fish resources in the Yangtze River, including the Chinese sturgeon, an endangered species. It is important to determine how the water temperature downstream shifts as the number of dams increases. As the total storage capacity continues to increase, will the impact of dams on water temperature reach a limit, or will the impact be moderated? To the authors’ knowledge, to date there has been no relevant research conducted on this topic; thus, the purpose of this paper is to address this gap. Through numerical simulation, the authors studied the influence of the dams on the thermal conditions in the river downstream and the causes of the variations in the downstream thermal conditions with the increasing number of dams.

## 2. Materials and Methods

Several objectives were addressed in this study. The one-dimensional model was verified using measured hydraulic data and water temperature data from 2014. A two-dimensional model was verified using measured water temperature data from a reservoir in 2013. The one-dimensional model was used to simulate the water temperature in the natural river reach. The water temperature in the reservoirs was simulated by the two-dimensional model. The effects of the dams on the water temperature in the river reach were analyzed. The effects of dam construction on the discharged water temperature in the reservoirs were studied. Based on the changing trend of downstream water temperature with the increase of the upstream total storage capacity, we predicted the trend of downstream water temperature when more dams are built in the future.

### 2.1. Study Area

**Figure 1:**
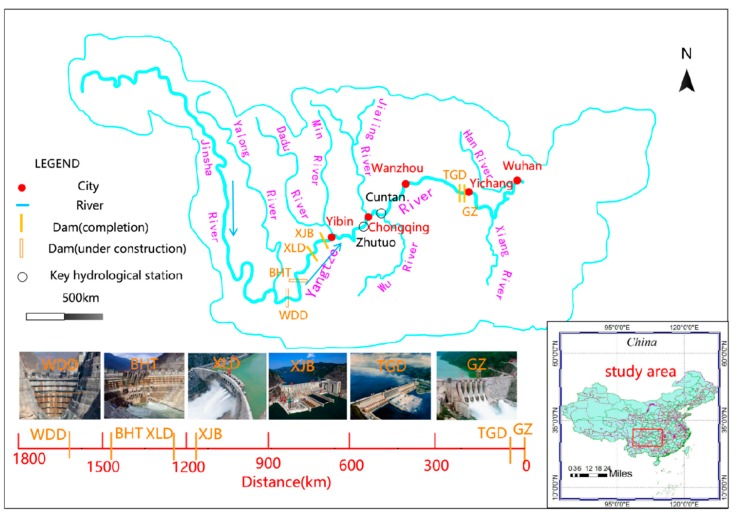
Locations of the dams in the Yangtze River. (GZ: Gezhouba, TGD: Three Gorges, XJB: Xiangjiaba, XLD: Xiluodu, BHT: Baihetan, and WDD: Wudongde).

The river reach between the Wudongde (WDD) and Gezhouba (GZ) dams on the Yangtze River was the study area, with a length of approximately 1800 km; shown in Figure 1. As shown in Table 1, since the 1980s several dams have been built on the main channel of the Yangtze River, including the Gezhouba (GZ), the Three Gorges (TGD), the Xiangjiaba (XJB), and the Xiluodu (XLD) dams. Additionally, two dams are under construction upstream of the XLD: Baihetan (BHT) and Wudongde (WDD). The Gezhouba is a relatively small reservoir at 47 m high and has a total storage capacity of 15.8 × 108 m^3^, and the others are all large reservoirs, with heights of more than 150 m and storage capacities of more than 50 × 108 m^3^.

The construction of these dams will change the flow of downstream river channels, and thermal stratification may occur in these reservoirs. The discharge water temperature from the reservoirs may be significantly different to that before the construction of the dams, and the downstream water temperature will vary. There are several tributaries in the study area (as shown in Figure 1), and they may also have an impact on the water temperature of the Yangtze River.

### 2.2. Model Description

The natural river remains between the XJB dam and Cuntan Station with no dam constructed, therefore the temperature changes only longitudinally, which can be simulated by a one-dimensional hydrodynamic model. The HEC-RAS model, which was developed by the U.S. Army Corps of Engineers Hydrology Engineering Center [33], was used to simulate the water temperature in the river reach from XJB to Cuntan. This hydrodynamic one-dimensional model can simulate hydrodynamics and water quality. The heat exchange at the air–water interface involves five components: net solar radiation, longwave radiation, reflective radiation, evaporation heat loss, and sensible heat transfer [34].

From Cuntan to Yichang, the TGD and GZ dams were built in succession. Thermal stratification may appear in the Three Gorges and Gezhouba reservoirs; the two-dimensional model can simulate the water temperature distribution in the longitudinal and vertical directions of the reservoirs. The water temperature of the TGD and GZ dams was simulated using the CE-QUAL-W2 model, which is a horizontally averaged two-dimensional hydrodynamic and water quality model that has been successfully applied to many river systems and was also developed by the U.S. Army Corps of Engineers [35]. CE-QUAL-W2 is based on the finite difference solution of laterally averaged equations of fluid motion, including: (a) hydrostatic pressure; (b) the free water surface; (c) horizontal momentum; (d) continuity; (e) constituent transport; and (f) the state equation of density. The inlet boundary conditions are the inflow discharge and water temperature at the reservoir tail, the outlet boundary condition is the outflow discharge, and the surface boundary conditions are based on environmental factors related to the reservoir water surface, including the air temperature, solar radiation, wind speed, humidity, and cloud cover [36,37].

### 2.3. Methods

The HEC-RAS model was verified by comparing the simulated water level and the water temperature with the measured data of the natural river between XJB and Cuntan. The calculation domain from XJB to Cuntan is 416 km, and it was divided into 120 sections with a cross section spacing of 0.5–2 km. The upstream boundary conditions included the outflow and the discharge water temperature of XJB. The downstream boundary condition was the water level at Cuntan, and the meteorological data from the Yibin Meteorological Station and the Chongqing Meteorological Station in 2014 were used as meteorological boundary conditions. In the hydrodynamic simulation with the HEC-RAS model, the value of the riverbed roughness was the key parameter, and the roughness in this paper ranged from 0.035 to 0.051.

The CE-QUAL-W2 model was verified using measured water temperature data from the TGD reservoir in 2013. The calculation domain of the TGD reservoir has a length of 600 km from Cuntan to the dam site. The simulated domain was divided into 938 (longitudinal) × 86 (vertical) rectangular grids with sizes of 300–700 m in the horizontal direction and 1 m in the vertical direction. The authors used the measured discharge water temperature data from November 2012 to June 2013 from the TGD reservoir to verify the accuracy of the water temperature model.

In this paper, some metrics are used to describe the seasonal and annual changes in the thermal regime, including annual average water temperature, extreme water temperatures, occurrence time of the highest and lowest water temperatures, the difference between the discharged water temperature from the reservoirs and the water temperature before the dam construction, and the seasonal delays in water temperature.

The change in the discharge water temperature in the XJB dam may have influenced the water temperature of the downstream river reach as well as the water temperatures of the TGD and GZ reservoirs; therefore, we analyzed this effect. To study the influence caused by the construction of dams on the downstream water temperature of the Yangtze River, we analyzed the influence in four construction periods (Table 2). The changes of the water temperature in the natural river channel and the changes of the inflow and discharge water temperature of TGD were analyzed to quantify the impact of the upstream dam construction on the downstream thermal regime. Guo W.X.’s research results showed that GZ had little effect on water release temperatures, and the discharge water temperature of TGD was essentially the same as that of the GZ [38]; therefore, we did not simulate the water temperature of GZ.

## 3. Results and Discussion

### 3.1. Model Validation

To verify the accuracy of the models, the hydrodynamics and water temperatures of the river reach from the XJB dam to Cuntan and the TGD reservoir were simulated and compared to the measured data. We considered the impact of tributaries (including the Min River, Tuo River, Jialing River, and Wu River) on the hydrodynamics and water temperature of the main channel of the Yangtze River.

**Figure 2:**
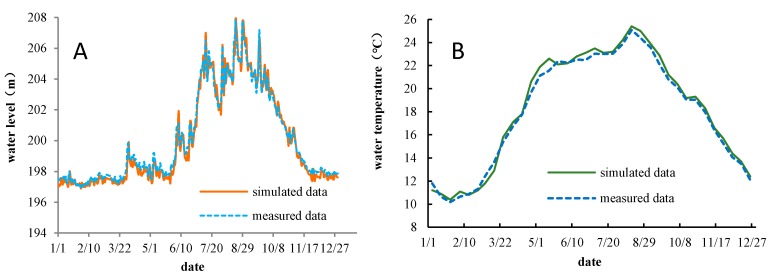
HEC-RAS model verification. (**A**) The water level at Zhutuo in 2014. (**B**) The water temperature at Cuntan in 2014.

Figure 2 shows that the simulated results of the HEC-RAS model were in good agreement with the measured data. The average difference between the simulated water level and the measured water level (simulated data minus measured data) at Zhutuo Station was 0.3 m, the difference in the water temperature range at Cuntan Station was −0.4~0.3 °C, the average difference was only 0.2 °C, and the simulated water temperature was lower than the measured values. The HEC-RAS model can accurately simulate the changes of hydrodynamic conditions in the study domain, and the heat balance under the influence of atmospheric heat exchange and tributaries inflow can also be effectively simulated.

**Figure 3:**
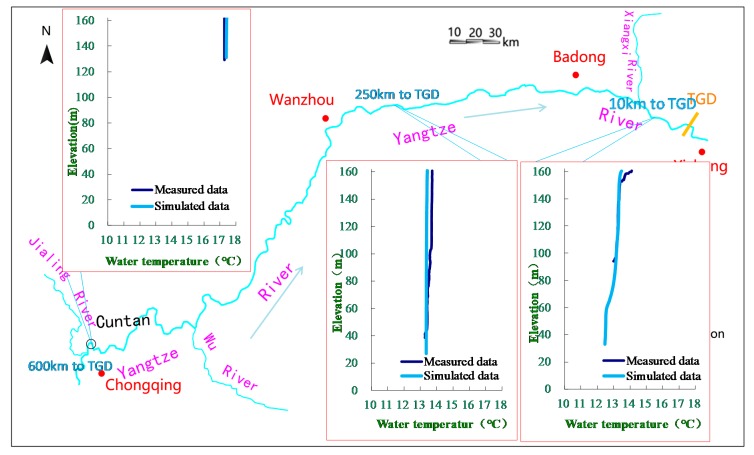
Comparison of simulated and measured water temperatures in the Three Gorges Reservoir on 28 March 2013.

**Figure 4:**
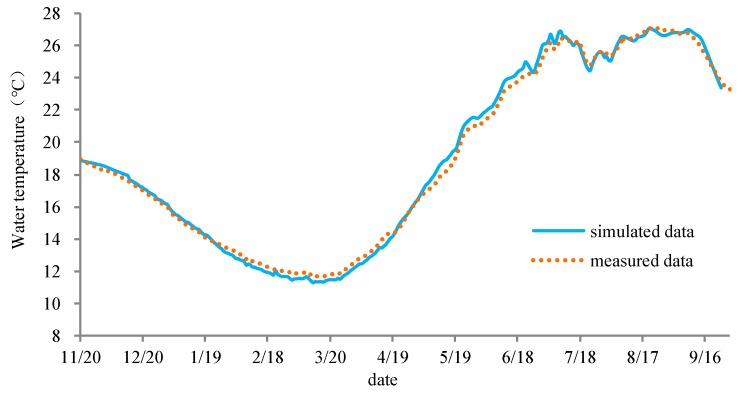
Comparison of the simulated and measured temperatures of water discharged from the TGD (from 20 November 2012 to 9 December 2013).

As shown in Figure 3, the simulated water temperature in the TGD reservoir was compared to the measured data on 28 March 2013. The results showed that the simulated water temperatures of the CE-QUAL-W2 model agreed well with the measured data. The distribution of the water temperature in the longitudinal and vertical directions were accurately simulated, and the average difference between the simulated data and measured data was only 0.2 °C. Figure 4 shows a comparison of the simulated discharged water temperatures of TGD and the measured data. The difference in the water temperature was −0.6 to 0.5 °C, the average difference was only 0.1 °C, and the simulated water temperature was lower than the measured values. The CE-QUAL-W2 model can effectively simulate the changes in water temperature, and the seasonal delay of water temperature due to reservoir impoundment.

### 3.2. The Discharge Water Temperature of XJB

**Figure 5:**
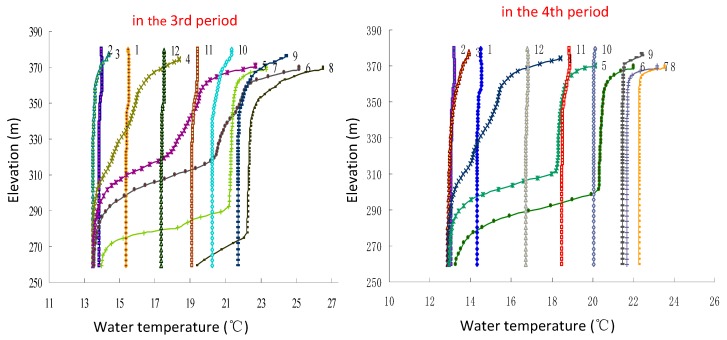
Vertical water temperature distribution in front of the XJB dam (1: January; 2: February; 3: March; 4: April; 5: May; 6: June; 7: July; 8: August; 9: September; 10: October; 11: November; 12: December).

**Figure 6:**
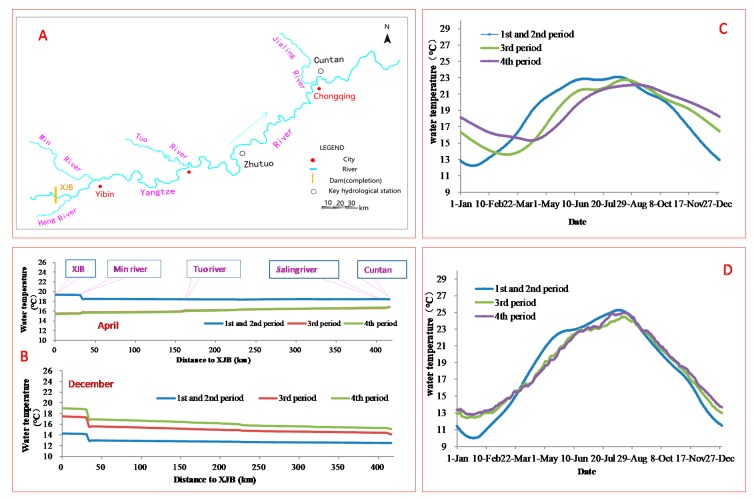
The simulated results of water temperature between XJB and Cuntan over the four periods. (**A**): The calculation domain. (**B**): The water temperature along the river between Xiangjiaba and Cuntan in April and December. (**C**): Daily water temperatures at XJB. The water temperatures in the 1st and 2nd periods are the average daily water temperatures measured from 1984 to 2012; the water temperatures in the 3rd and 4th periods are the simulated data. (**D**): Daily water temperatures at Cuntan; the water temperatures are simulated data. In the 1st and 2nd periods, XJB had not yet been completed, and there was no dam upstream of Cuntan; in the 3rd period, XJB and XLD had been completed. In the 4th period, XJB, XLD, BHT, and WDD had been completed.

After the construction of XJB and XLD (in the 3rd period), thermal stratification occurred in the XJB reservoir (as shown in Figure 5), and the discharge water temperature of XJB changed significantly compared to that before the dam was built (in the 1st and 2nd periods). For example, its range reduced, and the appearance time of the extreme value was delayed (as shown in Figure 6). The highest and lowest monthly average water temperatures before the construction of the dams were 22.9 °C (in August) and 12.4 °C (in January), respectively. After the XJB and XLD dams were constructed (in the 3rd period), the highest and lowest values were closer to the average water temperature, at 22.6 °C and 13.8 °C, respectively. The lowest water temperature appears in March, with a delay of two months compared to that in the 1st and 2nd periods. Moreover, from March to July, lower-temperature water was discharged; the average difference between the discharge water temperature in the 3rd period and that in the 1st period was 2.1 °C, and the maximum difference was 3.7 °C (in April). Higher-temperature water was discharged during November to January of the following year; the average difference was 2.8 °C, and the maximum difference was 3.3 °C in December.

The construction of the WDD and BHT dams (in the 4th period) had a further cumulative impact on the discharge water temperature of the XJB dam. The range of discharge water temperature was further reduced compared to that in previous periods, and the highest and lowest monthly average values were 22 °C (in August) and 15.5 °C (in April), respectively. Lower-temperature water was discharged from April to August, with time delayed by one month compared to that in the 3rd period; the average discharge water temperature was 2.6 °C lower than that in natural conditions, and there was a further decrease of 0.5 °C compared to that in the 3rd period. In May and June, the cumulative effect was the most severe, with further decreases of 1.8 °C and 1.5 °C compared to those in the 3rd period, respectively. From September to March of the next year, the discharge water temperature of XJB increased by an average of 1.3 °C compared to that in the 3rd period. In the 4th period, the impact time of higher-temperature water was also delayed by one month compared to that in the 3rd period.

### 3.3. Impact of Dams on the Inflow Water Temperature of TGD

There is a natural river reach with a total length of 416 km from the XJB dam to Cuntan with no dam. Figure 6 compares the water temperature in the four periods at XJB and Cuntan, and the simulated results show that, with the influence of climate and tributary inflow, the impact of dams on water temperature displayed a recovery at Cuntan (the tail of the Three Gorges reservoir). The higher-temperature water and lower-temperature water discharged from XJB flows along the river reach. After flowing through the 416 km channel, the impacts of dam constructions on water temperature were obviously weakened at Cuntan, but the effects did not completely disappear. The influence of the upstream dams on the water temperature will affect the water temperature of TGD. At Cuntan, the average difference between the water temperature in the 3rd period and that in the 1st period was 0.7 °C, with a reduction of 1.1 °C compared to the difference at XJB, and 61% of the impact caused by upstream dams was recovered. Notably, there was a recovery of 2.4 °C in April, and there was a recovery of 2.2 °C in December. After the construction of the BHT and WDD dams (in the 4th period), the average difference between the water temperature in the 4th period and the natural condition was 2.5 °C at XJB, which is greater than that in the 3rd period. Under the same meteorological and tributary inflow boundary conditions, the average difference in water temperature was only 1.3 °C at Cuntan, and 48% of the impact caused by the dams was mitigated.

### 3.4. The Discharge Water Temperature of TGD

**Figure 7:**
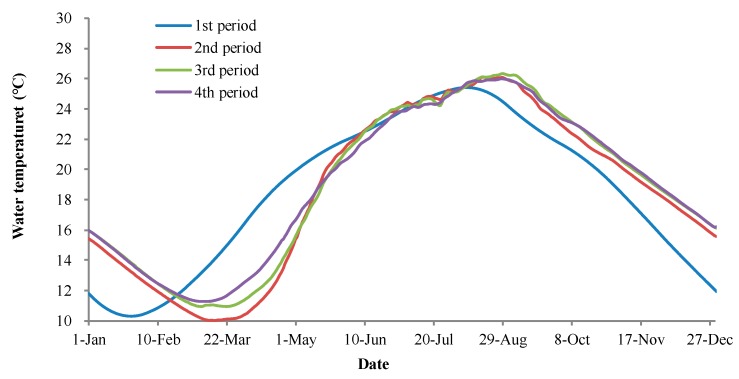
The discharge water temperature of TGD in each construction period.

According to the simulation results (as shown in Figure 7), the construction of the TGD had an obvious effect on the water temperature downstream, and the annual average discharge water temperature was 0.2 °C higher than that before the construction of TGD (in the 1st period), increasing from 18.4 °C to 18.6 °C. In some months (April, December, and January), the temperature varied by more than 3.0 °C. After the TGD reservoir went into operation, the water in the reservoir flowed more slowly than that in the natural river, and inflowing water at the reservoir tail now takes a longer time to flow to the dam; hence, there was a seasonal delay in the discharge water temperature of TGD compared to that before dam construction. In summer and autumn, the inflowing high-temperature water was stored in the reservoir, and it was discharged after several months. From September to February of the next year, the discharge water temperature of TGD was higher than that before construction of the dam (in the 1st period), and the average temperature increase was 2.4 °C; the maximum temperature increase was in January, when the variation reached 3.4 °C (10.7–14.1 °C). From March to July, the reservoir discharged lower-temperature water, which was stored in winter. The discharge water temperature was decreased by 2.0 °C on average, compared to that before the construction of TGD in these months, and the greatest variation was 4.6 °C in April. Due to the small inflow discharge in winter, the replacement rate of reservoir was small, and the low-temperature water that inflowed in January flowed to the dam site in March; the time when the lowest monthly average water temperature appears delayed by 2 months. In summer, because of the large inflow discharge, the replacement rate of reservoir was greater than that in spring; thus, there was no significant delay for the time when the highest monthly average appeared, which was observed in August in the 1st and 2nd periods.

The construction of the dams upstream in the lower reaches of the Jinsha River (WDD, BHT, XLD, and XJB) changed the discharge water temperature of XJB, although with the influence of climate and tributary inflow, the effect was mitigated to some extent at Cuntan, but more than 39% of the impact still exists, and it will affect the water temperature of TGD (as shown in Figure 5). Therefore, the influence of upstream dam construction on the water temperature of TGD was further studied in this paper.

In the 3rd period, with the construction of the XJB and XLD dams, the inflow water temperature of TGD changed (as shown in Figure 6D, daily water temperatures at Cuntan), and the inflow discharge also changed (as shown in Table 3). From January to May, the water levels in the upstream reservoirs (XJB and XLD) gradually decreased, and the outflow discharge was larger than the inflow discharge, which made the inflow discharge of TGD greater than that in the 2nd period, with an average increase of 523 m^3^/s. From June to September, the inflow discharge of TGD was less than that in the 2nd period, due to upstream reservoir impoundment. XJB and XLD maintained high water levels from October to December, and the inflow of TGD remained almost unchanged in the 2nd and 3rd periods. With the construction of BHT and WDD (in the 4th period), the changing trend of inflow discharge was similar to that in the 3rd period; from January to May, it was greater than that in 3rd period, but it was less than that in the 3rd period from June to October and remained almost the same from November to December.

Additionally, the influence of upstream dams (XJB, XLD, BHT, and WDD) on the XJB discharge water temperature was evident and continued to affect the water temperature of TGD. Under the combined influence of the inflow discharge and inflow water temperature, the discharge water temperature of the TGD changed significantly.

**Figure 8:**
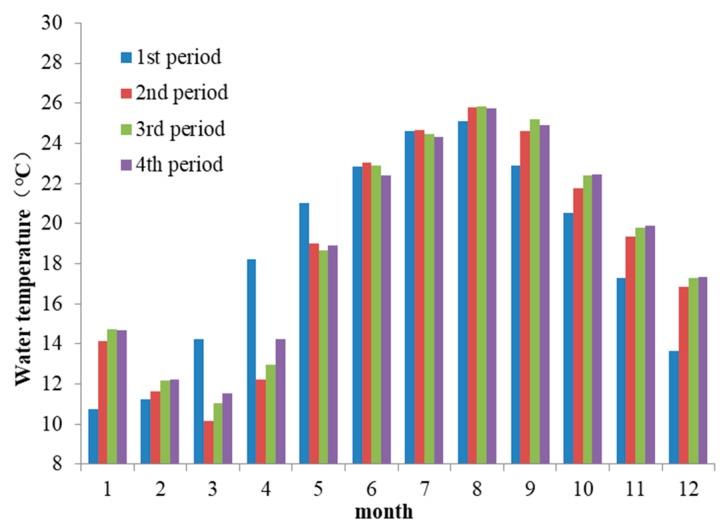
Monthly discharge water temperature of the Three Gorges dam.

As shown in Figure 8, in the 2nd period, the discharge water temperature of the TGD was lower than that before the TGD was built (in the 1st period), from March to July, and upstream dam construction mitigated this effect. In the 3rd period, the inflow discharge of the TGD increased from January to May, and replacement frequency was accelerated, which caused increases in the discharge water temperature of 0.6 and 0.8 °C, respectively, in March and April. The impact of the TGD construction on the thermal conditions in March and April was weakened compared to that in the 2nd period. In May, June, and July, affected by the low-temperature inflow water, the discharged water temperature of the TGD was lower than that in the 2nd period, with an average decrease of 0.3 °C. The average value of the discharge water temperature of the TGD from March to July was 0.2 °C higher than that in 2nd period. In the 4th period, after the construction of the BHT and WDD dams, the average discharged water temperature of the TGD increased further from March to July by 0.3 °C. In March, April, and May, the temperature was higher than that in the 3rd period, with increases of 0.5, 1.3, and 0.3 °C, respectively. In June, the discharge temperature was affected by the low-temperature water from the upstream dams, and it was 0.6 °C lower than that in 3rd period; this temperature did not change further in July.

After construction of the TGD, the discharge water temperature of the TGD was higher than that before the dam construction (in the 1st period) from September to February of the next year. Additionally, the construction of upstream dams had a further cumulative effect on the water temperature in these months. In the 3rd period, the discharge water temperature of the TGD increased by an average of 0.5 °C, and the variation ranged between 0.4 and 0.6 °C. In the 4th period, the discharge water temperature remained essentially unchanged compared to that in the 3rd period.

### 3.5. The Trend of Discharge Water Temperature at TGD in the Future

**Figure 9:**
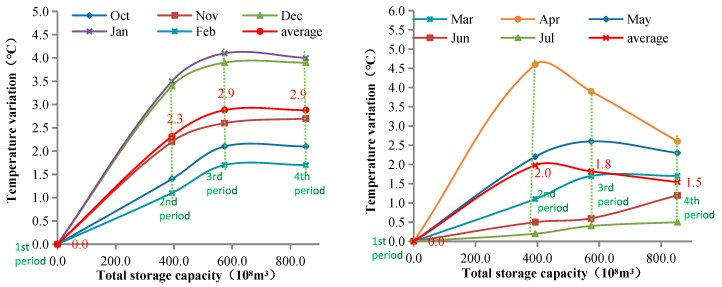
The difference between the discharged water temperature of the TGD and the natural water temperature. The total storage capacity is the sum of the storage capacity of each reservoir. In the 1st period, the total storage capacity is 0. In the 2nd period, the total storage capacity is 393 × 10^8^ m^3^. In the 3rd period, the total storage capacity is 574 × 10^8^ m^3^. In the 4th period, the total storage capacity is 853 × 10^8^ m^3^.

In the future, more and more dams will be built upstream of the TGD. How will the discharge temperature of the TGD be affected after more dams are constructed? We need to conduct research in this area, because there are many aquatic organisms sensitive to water temperature in the downstream river reaches of the Three Gorges. We therefore performed corresponding analyses and predictions. The relationship between the variation (compared to that in the 1st period) of discharge temperature of the TGD and the total storage capacity of the Yangtze River is shown in Figure 9. The results showed that from October to February of the following year, with the construction of the TGD (in the 2nd period), XJB, and XLD dams (in the 3rd period), the discharge water temperature of the TGD increased as the total storage capacity increased. With the construction of the BHT and WDD (in the 4th period), there was no obvious cumulative effect; the discharge water temperature did not increase further, and the average temperature variation (the discharge water temperature of the TGD minus the natural water temperature at TGD dam) remained at 2.9 °C. From March to July, as the storage capacity increased, the average discharge water temperature increased, and the temperature variation decreased. After TGD operation began (in the 2nd period), the temperature variation was the greatest, and the average variation was 2.0 °C. In the 3rd period, the average temperature variation was reduced to 1.8 °C, and it was further reduced to 1.5 °C in the 4th period. It is important to note that the discharge water temperature of the TGD in June and July decreased with the increase of the total storage capacity, and the temperature variation increased. In the 3rd period, the variation increased by 0.1 and 0.2 °C, respectively, and it further increased by 0.5 and 0.2 °C, respectively.

We can conclude that, from October to February, the discharge water temperature of TGD increased with the increase in the storage capacity of the Yangtze River, but this trend was not continuous; when the total storage capacity reached a certain level, the water temperature did not increase further. If the total storage capacity continues to increase in the future, there will be no significant impact on the discharge water temperature during these months. The discharge water temperature of the TGD was lower than that before the construction of the dam from March to July, and the discharge water temperature trend was different from that between October and February. With the construction of upstream dams, the temperature variation will significantly decrease in March, April, and May, and the variation in the discharge water temperature of the TGD will increase slightly in June and July. Continuous dam construction upstream will weaken the impact of the low-temperature water discharged from TGD.

## 4. Conclusions

The construction of multiple dams had different cumulative impacts on the water temperature downstream. With the construction of Xiangjiaba and Xiluodu, the range of the discharge water temperature of XJB was reduced, and the occurrence of extreme water temperatures was delayed. From March to August, lower-temperature water (compared to the water temperature before dam construction) was discharged, and higher-temperature water was discharged from November to January. Due to the influence of climate and tributaries, the impact of upstream dams was obviously mitigated by more than 48% at Cuntan, the tail of the Three Gorges reservoir, but it was still observable.

With the construction of the Three Gorges dam, its annual average discharge water temperature increased by 0.2 °C compared to the natural water temperature. From September to February of the next year, the discharge water temperature (in the 2nd period) was 2.1 °C higher than that before the construction of the dam (in the 1st period). With the construction of Xiangjiaba and Xiluodu (in the 3rd period), the temperature variation (the discharge water temperature of the TGD minus the natural water temperature at TGD dam) increased to 2.7 °C. After the Baihetan and Wudongde dams are constructed in 2021 (in the 4th period), we believe there will be no further temperature increases from September to February of the next year. In spring (March–May), the water temperature discharged from the Three Gorges (in the 2nd period) was 3.1 °C lower than that in the 1st period. As the number of dams increased, the discharge water temperature from March to May increased conversely; it seemed that the cumulative effect, even the negative cumulative impact, decreased with the increase of the upstream total storage capacity.

## Figures and Tables

**Table 1:** The main characteristics of the six dams of interest and their corresponding acronyms.

Dam	Acronym	Completion Time	Storage Capacity (108 m^3^)	Distance from Gezhouba (km)
Gezhouba	GZ	1982	16	0
Three Gorges	TGD	2009	393	38
Xiangjiaba	XJB	2012	52	1100
Xiluodu	XLD	2014	127	1250
Wudongde	WWD	2020 (planned)	74	1650
Baihetan	BHT	2021 (planned)	206	1450

**Table 2:** The four periods of dam construction on the Yangtze River.

Period	Years	Dams				
1st	Before 2003	GZ				
2nd	2009–2012	GZ + TGD	XJB	XLD		
3rd	2014–2020	GZ + TGD	XJB	XLD		
4th	After 2020	GZ + TGD	XJB	XLD	BHT	WDD

**Table 3:** Monthly inflow discharge and the number of replacements for the Three Gorges Reservoir. The number of replacements is defined as the ratio of total inflow to reservoir capacity per month.

Month	Inflow Discharge (m^3^/s)			Number of Replacements		
	2nd Period	3rd Period	4th Period	2nd Period	3rd Period	4th Period
January	4474	4985	5246	0.35	0.39	0.41
February	4155	4700	5418	0.31	0.35	0.41
March	5202	5738	6389	0.46	0.51	0.57
April	6723	7371	8265	0.60	0.66	0.74
May	11,085	11,462	12,802	1.19	1.23	1.37
June	15,436	14,747	14,718	1.98	1.89	1.89
July	30,133	30,106	27,156	3.72	3.71	3.35
August	20,703	20,758	20,596	2.61	2.62	2.60
September	20,323	18,523	18,185	1.97	1.80	1.77
October	14,536	14,495	13,883	1.13	1.12	1.07
November	8765	8790	8670	0.62	0.62	0.61
December	5570	5538	5881	0.41	0.41	0.43
